# The Story of the Insane from Year to Year

**Published:** 1897-09-18

**Authors:** 


### THE STORY OF THE INSANE FROM YEAR TO YEAR.

Tenth Series.

### DORSET COUNTY ASYLUM.

This asylum contained 611 patients on January 1st, 1896, and 710 on December 31st. There were 191 first admissions, 22 readmissions, 46 recoveries, 7 discharged relieved, and 42 deaths. The recoveries stand at 31·29 per cent. of the admissions, and the deaths at 6·19 per cent. on the average number resident. Of the deaths, 14 were caused by cerebral and spinal diseases, 4 by consumption, and 1 by pneumonia. Various moral causes, such as domestic trouble and religion, caused the insanity in 37 of the year's admissions, intemperance in drink in 12, and hereditary influence in 38. Dr. Macdonald has under his care 104 out-county cases, and he says with much truth that such patients constitute a disturbing element. "If not abusive, they are discontented; if not fault-finding, they are grumbling; and if not explaining the Lunacy laws they are writing volumes on their grievances." The block for private patients was completed during the year, and the private patients have increased from 27 to 75; and it is practically certain that the visitors could obtain double that number if they had room for them. When discussing the causation of insanity, Dr. MacDonald gives a prominent place to domestic trouble, but he shows that hereditary influence is the most potent of all causes, and he says these cases will continue to swell the annual admissions until there is some check to the marriage of persons predisposed to insanity. He does not tell us what that check is to be. Nursing lectures are given by Drs. Davidson and Hanbury, and to those of the staff successful at the examinations the visitors grant certificates and extra pay. We congratulate Nurse Pomeroy on having obtained her pension after 16 years' service. The visitors say that the medical superintendent "continues to merit their highest approval in his effective and successful management of the asylum." The average weekly cost of maintenance was 8s. 8⅝d. per head. Out-county patients pay 14s., and private patients from 10s. to £2 2s. a week.

### SALOP AND MONTGOMERY ASYLUM.

Here the numbers rose during the year from 810 to 834. There were 144 admissions, 36 readmissions, 66 recoveries, 14 discharged relieved, and 77 deaths. The recoveries stand at 36·66 per cent. on the admissions, and the deaths at 9·34 per cent. on the average number resident. Hereditary influence alone, or in combination with other causes, accounts for 34 of the year's admissions, and drink for 19, while 47 are said to be of unknown causation. Cerebral and spinal diseases caused death in 34 instances, consumption in 11, and pneumonia in 4. The Commissioners say, "The proportion of post-mortem examinations continues to be very unsatisfactory," and Dr. Strange said he did all he "could to get the rule as to post-mortem examinations altered, and as the Commissioners were unable to do so," he thinks they might either have omitted the remark or extended it to show the fault did not lie with him. Dr. Strange also disagrees with the Commissioners' way of calculating the proportion of attendants to patients. The visitors say that "the manner in which the superintendent and the other officers of the institution have performed their duties is deserving of praise.' The average cost of maintenance is 7s. 5½d. per head per week. Private patients pay 15s.

### HEREFORD COUNTY ASYLUM.

The twenty-fifth annual reports tells us that 393 patients were in residence on January 1st, 1896, and that the numbers had risen to 400 on the last day of the year. There were 45 admissions, 25 readmissions, 18 recoveries, 22 discharged relieved, and 23 deaths. Calculated in the usual way the recoveries stand at 25·7 per cent., and the deaths at 5·7 per cent. Both of these percentages are considerably below the average of kindred institutions, and indeed below the average of the Hereford Asylum during the previous 24 years. No definite conclusion can be drawn from one year's statistics of insanity, or even from many years' statistics, because the conditions are never quite the same in any two counties in England. Of the deaths 10 were caused by cerebral and spinal diseases, and 2 to pneumonia, pulmonary consumption being entirely absent from the list. Ten of the year's admissions owed their insanity to various moral causes, 9 to intemperance in drink, and 12 to hereditary tendency. The admissions seem to have been of a very unfavourable type, for Dr. Morrison says that not more than 20 per cent. offered any hope of recovery; and when commenting on the fact that the workhouses are being emptied of their imbeciles and the asylums being filled with them, he gives it as his opinion that some alteration should be made in the procedure of admission to asylums, or that a different class of institution should be created; otherwise recoverable cases do not get that individual attention necessary to recovery. If this be true of a small asylum like Hereford what must it be in those places where 1,500 or 2,000, or more even than that are under the same roof? But while condemning large asylums and deploring the existence of a law which permits the building of any above 1,000 beds, we do not think that the mixture of various forms of insanity is so prejudicial to individual attention as Dr. Morrison supposes. It may be that the presence of 20 demented patients in a ward are not the best possible surroundings for a person suffering from acute insanity, but surely they are better than a like number of acute cases. The asylum is now full, and twenty patients are to be boarded out at Grove Hall and Norwich; but plans for the enlargement of the asylum have been prepared. Dr. Chapman resigned the office of medical superintendent, and was granted a pension. We regret that the resignation was owing to ill-health. Mr. Smith, head attendant, and Mrs. Smith, the housekeeper, also retired on their pensions. After twenty-five years' service they deserve a rest, and we hope they may long enjoy their well-earned pensions. The average weekly cost per head was 8s. 9¼d.

### WILTS COUNTY ASYLUM, DEVIZES.

On January 1st, 1896, there were 744 patients in residence, and on the last day of the year the numbers had risen to 789. There were 133 first admissions, 26 readmissions, 56 recoveries, 9 discharged relieved, and 47 deaths. Calculated on the admissions, the recoveries stand at 35·2 per cent., and calculated on the average number resident the deaths stand at 6·1 per cent., the former being slightly below the county asylum average, and the latter decidedly below it. Of the deaths 12 were caused by cerebral and spinal diseases, 4 by consumption, and 3 by pneumonia. Mental anxiety and domestic troubles account for the insanity in 23 of the year's admissions, intemperance in drink for 9, and hereditary tendency for 34. Mr. Bowes has compiled a very interesting report. When referring to the large increase in the number of patients he says that no explanation is forthcoming, but he thinks that various factors are at work leading up to it—for instance, the low death-rate, the fact that lunatics are more readily sent to asylums than was formerly the case, and also that purely agricultural counties produce a higher proportion of insanity than manufacturing districts. The latter fact has long been recognised, and it would seem certain that wherever wages are low insanity is high. Although additions have lately been made to the asylum there are only 34 vacant beds, being just about sufficient for the probable increment of 1897, so that once more additions are called for. When speaking of the resignations among the nursing staff, Mr. Bowes says, "It is a pity nurses with odds and ends of experience are sought after and so readily engaged by those in authority in certain asylums." It is, indeed, a great pity, and until the practice is abandoned we shall never have really "settled" staffs in our asylums; but Mr. Bowes is no doubt aware, and attendants and nurses ought to be aware, that such attendants and nurses are not received in the best asylums, and when they do succeed in obtaining appointments their success is rarely a good thing for themselves or for the asylum they are taken in at. The visitors say they have "again to express their entire satisfaction with the admirable manner in which the medical superintendent manages the asylum." The weekly rate of maintenance is 8s. 7½d. per head.

